# MicroRNA profiling analysis of developing berries for ‘Kyoho’ and its early-ripening mutant during berry ripening

**DOI:** 10.1186/s12870-018-1516-x

**Published:** 2018-11-16

**Authors:** Da-Long Guo, Qiong Li, Wen-Qing Lv, Guo-Hai Zhang, Yi-He Yu

**Affiliations:** College of Forestry, Henan University of Science and Technology, Luoyang, 471023 Henan Province China

**Keywords:** Grape, Early ripening, miRNA, ROS

## Abstract

**Background:**

‘Fengzao’ is an early-ripening bud mutant of ‘Kyoho’, which matures nearly 30 days earlier than ‘Kyoho’. To gain a better understanding of the regulatory role of miRNAs in early-ripening of grape berry, high-throughput sequencing approach and quantitative RT-PCR validation were employed to identify miRNAs at the genome-wide level and profile the expression patterns of the miRNAs during berry development in ‘Kyho’ and ‘Fengzao’, respectively.

**Results:**

Nine independent small RNA libraries were constructed and sequenced in two varieties from key berry development stages. A total of 108 known miRNAs and 61 novel miRNAs were identified. Among that, 159 miRNAs identified in ‘Fengzao’ all completely expressed in ‘Kyoho’ and there were 10 miRNAs specifically expressed in ‘Kyoho’. The expression profiles of known and novel miRNAs were quite similar between two varieties. As the major differentially expressed miRNAs, novel_144, vvi-miR3626-3p and vvi-miR3626-5p only expressed in ‘Kyoho’, vvi-miR399b and vvi-miR399e were down-regulated in ‘Fengzao’, while vvi-miR477b-3p up-regulated in ‘Fengzao’. According to the expression analysis and previous reports, miR169-NF-Y subunit, miR398-CSD, miR3626-RNA helicase, miR399- phosphate transporter and miR477-GRAS transcription factor were selected as the candidates for further investigations of miRNA regulation role in the early-ripening of grape. The qRT-PCR analyses validated the contrasting expression patterns for these miRNAs and their target genes.

**Conclusions:**

The miRNAome of the grape berry development of ‘Kyoho’, and its early-ripening bud mutant, ‘Fengzao’ were compared by high-throughput sequencing. The expression pattern of several key miRNAs and their target genes during grape berry development and ripening stages was examined. Our results provide valuable basis towards understanding the regulatory mechanisms of early-ripening of grape berry.

**Electronic supplementary material:**

The online version of this article (10.1186/s12870-018-1516-x) contains supplementary material, which is available to authorized users.

## Background

Grape (*Vitis vinifera* L.) is an important fruit crop in the world with a high nutritional value for fresh berry consuming and wine-making. The proper ripening time point is vital either for table or wine grape. Fruit ripening is a complex processes involved enormous physiological and molecular changes in the color, flavor, quality, and the regulations of gene expressions. At present, most of our knowledge about the genetic and molecular control of fruit ripening are from the model fruit tomato with the various ripening-related mutants [1, 2].

Studies have been conducted with the ripening mutants from different fruit crops to explore the molecular mechanisms of fruit ripening, such as in citrus [3], pear [4], and grape [5]. We have found an early-ripening bud mutant of ‘Kyoho’, ‘Fengzao’, which matures earlier 30 days than ‘Kyoho’ [6]. The phenological, physiological and molecular differences between ‘Fengzao’ and ‘Kyoho’ have been investigated in the previous studies [6–10]. Histological and molecular analysis showed that their genetic background are highly uniform [9]. RNA-Seq analysis showed that the main differentially expressed genes between ‘Fengzao’ and ‘Kyoho’ are ROS (Reactive Oxygen Species) related genes [8]. More proofs need to be explored to clarify the early-ripening mutant mechanism of ‘Fengzao’.

MicroRNAs (miRNAs) are a class of ~ 22 nt endogenous non-coding RNAs and negatively regulate the expressions of target genes through mRNA degradation or translational inhibition of target mRNAs [11]. Plant miRNAs have been extensively analyzed in tomato [1], sweet orange [3] and melon [12], etc. miRNAs were confirmed to be involved in multiple processes, such as stress response [13], fruit development [14, 15] and floral bud development [16], etc.

Recently, some studies have suggested that miRNAs play an important role in various fruit development and ripening process including pear [17], *Lycium barbarum* [18], *Arabidopsis* [19], sweet orange [3] and melon [12], etc. Karlova et al. [20] reported that *CNR* and *SIAP2a* were actively modulated by miR156/157 and miR172 during tomato ripening. Gao et al. [1] identified several miRNAs, which differentially expressed during fruit development in tomato *RIN* mutant. Saminathan et al. [15] showed that miR156, miR156a, miR159a, miR159b, and miR319b were upregulated during the later stages of pomegranate fruit development. There are a relatively large number of grape miRNAs deposited in miRBase Release21 and their expression profiles have been analyzed in developing berries [21–23]. Some reports suggested that the mutations of the mutant have modified the regulation of miRNAs expressions between the mutant and wild genotypes [24–26]. However, the mechanism underlying the miRNA-mediated regulation of early-ripening of grape berry is largely unknown. Therefore, identification and characterization of miRNAs in grape early ripening bud mutant may provide new insights for grape berry ripening process.

To gain a better understanding of the regulatory role of miRNAs in early-ripening of grape berry, the miRNA profiles during the berry development of ‘Kyoho’ and its early-ripening bud mutant, ‘Fengzao’ were investigated using an Illumina HiSeq 2000 platform. The differentially expressed miRNAs during the berry development of ‘Fengzao’ and ‘Kyoho’ were identified, and the corresponding target genes were predicted. In addition, the expression patterns of several miRNAs and their target genes were further examined by qRT-PCR analyses. The results provided some insights into the regulatory roles of miRNAs during grape berry development, and the data presented here will lay a foundation for future studies of grape early-ripening.

## Results

### Small RNA profiles of grape berry development

Nine independent small RNA libraries were generated from key berry development stages of ‘Fengzao’ and ‘Kyoho’ (Table 1). The sampling time points were selected based on our earlier researches which indicated that these berry development stages are key to reveal the significant transcriptional and metabolic varitions between ‘Fengzao’ and ‘Kyoho’ [7, 8]. For samples of ‘Fengzao’, 11,524,331 reads were obtained on average per sample (Additional file 1: Table S1). While for ‘Kyoho’, the averages are 12,717,569 reads (Additional file 1: Table S1). After filtering the low-quality reads, adapter contaminates, reads with ploy Ns, clean reads ranging from 10,245,269 to 12,987,903 were obtained per sample. Then, reads of length < 18 were excluded, resulting in 9,103,202 reads on average. Subsequntly, more than 66% of the screened reads were successfully mapped to grape reference sequence.

**Table 1:** The sampling time and corresponding development stages of grape berries in this study

Development stage	Sampling date		Sampling date		Stage code	Characteristics
	Fengzao	Code	Kyoho	Code		
E-L33	6.17	FZ1	6.27	KY1	I	hard green berries
			7.4	KY2	II	
E-L34	6.27	FZ2	7.16	KY3	III	starting to soften
E-L35	7.4	FZ3	7.23	KY4	IV	véraison
E-L37	7.11	FZ4	8.3	KY5	V	Berries not quite ripe

Samples were collected in 2016

**Fig. 1:**
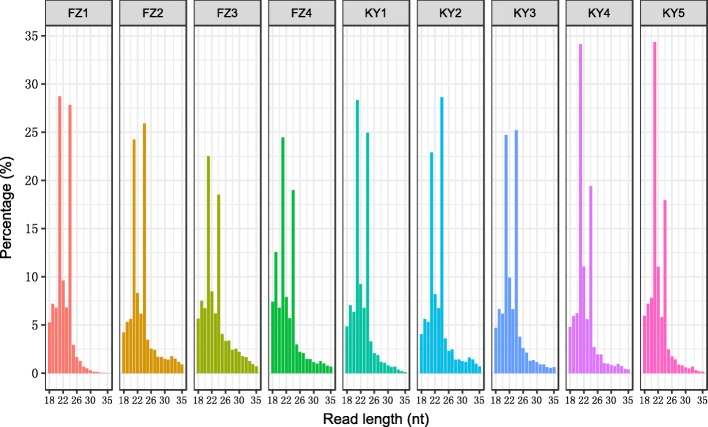
Length distribution of unique sRNAs in grape berry libraries. Y-axis represents the percentages of small RNA identified in each sample. X-axis represents the length of small RNA. FZ1–4 and KY1–5 represent the samples from different berry development stages as indicated in Table 1. The sample codes are the same as below

The length of the small RNA (sRNA) ranged from 18 to 35 nt were counted (Fig. 1). The dominant and abundant sRNA length were 21 and 24 nt regardless of the development stages and whatever in ‘Kyoho’ or ‘Fengzao’ (Fig. 1). The 21 nt sRNAs were the most abundant, followed by 24 nt sRNAs. The amount of 21 nt and 24 nt accounted for 60% of all the sRNA reads. However, the number of 24 nt sRNAs is a little more than that of 21 nt at the sample of FZ2 and KY2.

The size distribution of 20, 22 and 23-nt redundant reads has no obvious difference between ‘Kyoho’ and ‘Fengzao’. From Fig. 1, it could be observed that the length distribution profiles of sRNAs were very similar between ‘Kyoho’ and ‘Fengzao’ during the whole berry development stages. The common and specific sRNAs were compared between any two libraries, showing that more than 60% of the total sRNAs were shared.

The sRNAs were analyzed using the blastn and blastall alignments against Genbank and Rfam databases to filter out the known non-coding RNAs: snoRNAs, snRNAs, tRNAs, and rRNAs. All of nine libraries showed similar compositions of non-conding RNA types (Table 2). The number of reads differed in each category. Particularly, the proportion of repeat and NAT (natural antisense short interfering RNA) specific reads was high (10.6 and 10.7%) among the unique reads. Most abundant non-coding RNA class was rRNA for unique reads with the mean value of 8.21%, and other RNAs had quantity with the average of 0.16% (snRNA) and 0.58% (snoRNA) (Table 2). The remaining sequences were examined to identify miRNAs, and the un-annotated sequences were used for novel miRNAs identification. Afterwards, the known miRNA is 4.65%, novel miRNA is 2.39% on average.

**Table 2:** Summary of small RNA sequencing data and annotation after alignment to GenBank and Rfam in nine libraries

	FZ1	FZ2	FZ3	FZ4	KY1	KY2	KY3	KY4	KY5
Total	5,511,599	6,292,055	6,206,300	6,835,422	7,080,977	6,531,036	6,498,722	6,944,941	6,876,953
known_miRNA	293,764 (5.33%)	256,923 (4.08%)	195,676 (3.15%)	240,682 (3.52%)	304,883 (4.31%)	220,877 (3.38%)	288,939 (4.45%)	468,847 (6.75%)	473,968 (6.89%)
rRNA	401,277 (7.28%)	461,547 (7.34%)	819,911 (13.21%)	555,173 (8.12%)	490,108 (6.92%)	408,859 (6.26%)	614,342 (9.45%)	530,932 (7.64%)	526,145 (7.65%)
tRNA	2 (0.00%)	1 (0.00%)	0 (0.00%)	1 (0.00%)	1 (0.00%)	2 (0.00%)	1 (0.00%)	0 (0.00%)	0 (0.00%)
snRNA	6216 (0.11%)	7435 (0.12%)	8612 (0.14%)	7961 (0.12%)	8801 (0.12%)	6698 (0.10%)	6213 (0.10%)	7397 (0.11%)	7916 (0.12%)
snoRNA	29,043 (0.53%)	45,448 (0.72%)	25,971 (0.42%)	29,690 (0.43%)	55,917 (0.79%)	47,378 (0.73%)	52,260 (0.80%)	31,061 (0.45%)	28,381 (0.41%)
repeat	705,647 (12.80%)	696,292 (11.07%)	510,942 (8.23%)	605,622 (8.86%)	759,921 (10.73%)	750,842 (11.50%)	743,158 (11.44%)	715,266 (10.30%)	723,924 (10.53%)
NAT	609,654 (11.06%)	640,231 (10.18%)	620,372 (10.00%)	636,276 (9.31%)	664,891 (9.39%)	647,042 (9.91%)	688,849 (10.60%)	866,898 (12.48%)	935,099 (13.60%)
novel_miRNA	123,239 (2.24%)	150,151 (2.39%)	124,871 (2.01%)	181,311 (2.65%)	171,565 (2.42%)	127,118 (1.95%)	168,580 (2.59%)	166,758 (2.40%)	200,238 (2.91%)
TAS	16,101 (0.29%)	13,801 (0.22%)	11,967 (0.19%)	14,884 (0.22%)	14,451 (0.20%)	13,756 (0.21%)	23,823 (0.37%)	17,509 (0.25%)	21,585 (0.31%)
Exon_sense	124,655 (2.26%)	112,184 (1.78%)	96,842 (1.56%)	110,968 (1.62%)	125,125 (1.77%)	101,251 (1.55%)	154,621 (2.38%)	152,438 (2.19%)	160,113 (2.33%)
Exon_antisense	113,779 (2.06%)	84,968 (1.35%)	65,792 (1.06%)	79,570 (1.16%)	101,249 (1.43%)	80,722 (1.24%)	146,666 (2.26%)	124,805 (1.80%)	125,799 (1.83%)
Intron_sense	50,417 (0.91%)	67,408 (1.07%)	56,941 (0.92%)	72,555 (1.06%)	59,421 (0.84%)	67,508 (1.03%)	70,087 (1.08%)	76,390 (1.10%)	85,546 (1.24%)
Intron_antisense	29,843 (0.54%)	30,098 (0.48%)	30,491 (0.49%)	30,652 (0.45%)	32,891 (0.46%)	34,738 (0.53%)	40,122 (0.62%)	47,752 (0.69%)	57,420 (0.83%)
Un_annotated	3,007,962 (54.58%)	3,725,568 (59.21%)	3,637,912 (58.62%)	4,270,077 (62.47%)	4,291,753 (60.61%)	4,024,245 (61.62%)	3,501,061 (53.87%)	3,738,888 (53.84%)	3,530,819 (51.34%)

rRNA/snRNA/snoRNA/tRNA considered; TAS trans-acting small interfering RNA, NAT natural antisense short interfering RNA

### Identification of known and novel miRNAs in grape berry

**Fig. 2:**
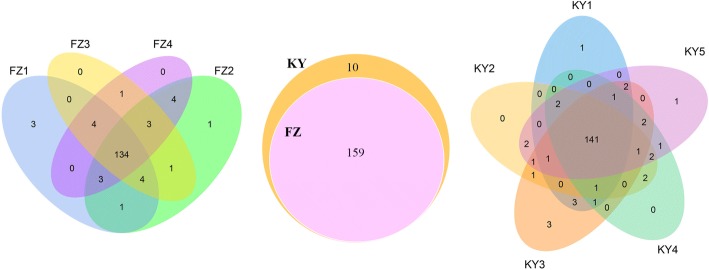
Venn diagram showing miRNAs identified in this study. The left and right showed the miRNAs profile between different berry developmental stages of ‘Fengzao’ and ‘Kyoho’, respectively. The middle showed the miRNAs profile shared between ‘Fengzao’ and ‘Kyoho’

After further sequence analysis, a total of 159 miRNAs were identified in ‘Fengzao’, among which, known miRNAs were 100. While for ‘Kyoho’, total and known miRNAs were 169 and 108, respectively (Additional file 2: Table S2). The sequence of all known and novel miRNAs were shown in Additional file 3: Table S3. For ‘Fengzao’, 134 sRNAs were common among four development stages (Fig. 2). While for ‘Kyoho’, it was 141. Among all these miRNAs, 159 were shared between ‘Kyoho’ and ‘Fengzao’, 10 was specific for ‘Kyoho’: novel_139, novel_144, vvi-miR156a, vvi-miR156e, vvi-miR171h, vvi-miR3626-3p, vvi-miR3626-5p, vvi-miR845a, vvi-miR845c, vvi-miR845d. The expression levels of most specific miRNAs in ‘Kyoso’ were low except vvi-miR3626-3p and vvi-miR3626-5p. The target genes predicted by psRobot softwar were shown in Additional file 4: Table S4. Most of the target genes were related to activities of ATPase, Zinc finger, Leucine-rich repeat, NADP-dependent oxidoreductase domain, Protein kinase, expecially Serine/threonine-protein kinase, ATP binding sites, Phosphorylation, etc.

In this study, these known miRNAs could be classified into 35 conserved miRNA families (Additional file 5: Figure S1; Additional file 6: Table S5). Most of the conserved families were present across the different plants species (Additional file 5: Figure S1). The miRNAs composition in each family identified in this study were similar to that of *Malus domestica* (Additional file 5: Figure S1), indicating high conservation of miRNA families and high homology between two species. Some known but less-conserved miRNAs were also found in this study (MIR845_2, MIR3630, MIR3631, MIR845_3, MIR2950). Among these, MIR169_2 were the largest represented families with 11 members, followed by MIR399 and MIR171_1, with six and five members, respectively. Of the remaining families, most of them comprised two to four members. Furthermore, various family members from the same miRNA family showed diverse expression levels. For example, the number of MIR396 family members reads ranged from1839–29,792, while for MIR169_2 family members, reads ranged from 2 to 987. For vvi-miR399d (2–37 reads) and vvi-miR399a (73–645 rerads), both of them belong to the same famlily MIR399, but the expression levels showed largely variable.

Nucleotide bias analysis of these known miRNAs revealed that uridine (U) is the most common preferred nucleotide at the first position of the 5′end (> 85%); while adenine (A; ~ 40%) was the most common nucleotide at both the 10th and 11th nucleotide matched to the cleavage site of the targets (Additional file 7: Figure S2A). For 18- to 28-nt miRNAs, the base bias at the first position from the 5′ end had a strong preference for U. Nucleotides A and G predominately occupied the first position base bias for the 29- and 30-nt miRNAs, respectively (Additional file 7: Figure S2B). Nucleotide U was preferred more than 80% of the time as a first base for 18- to 30-nt miRNAs.

After removing conserved and known miRNAs, the remaining sequences were used to predict novel miRNAs according to the sequence similarity and the formation of a stable stem-loop structure in the precursor. Finally, 59 and 61 potential novel miRNAs were predicted from ‘Fengzao’ and ‘Kyoho’, respectively (Additional file 2: Table S2). Most of predicted novel miRNAs expressed both in ‘Fengzao’ and ‘Kyoho’, except novel_139 and novel_144, which only expressed in ‘Kyoho’.

### Prediction of putative target genes for known and novel miRNAs

A total of 2162 unigenes were predicted to be targets of 105 miRNAs using PsRobot software (Additional file 4: Table S4). A relatively high proportion of target genes were annotated as transcription factors (TFs) and protein-like domains. A number of miRNAs had multiple putative target genes, the numbers of targets for each ranging from 1 (vvi-miR168, vvi-miR3627-3p) to 331 (for vvi-miR3629a-5p), only 24 miRNAs below 4 targets, suggesting that these miRNAs may have diverse biological functions. In contrast, several putative target genes were targeted by multiple miRNAs with up to 14 miRNAs for a hypothetical protein (VIT_11s0103g00390), on average 1.44. Noticeably, only 1 out of the putative 61 novel miRNAs (novel_141) was successfully predicted to target 192 unigenes.

**Fig. 3:**
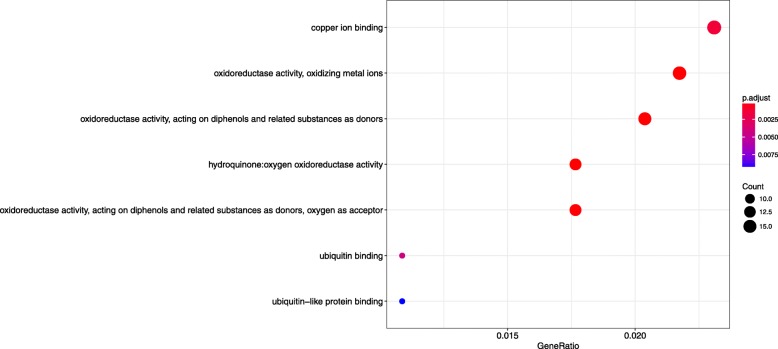
Scattergram of overrepresented GO terms (*P* < 0.05) in molecular function categories from GO enrichment analysis of miRNA targets gene of all the miRNAs identified in this study using ClusterProfiler. Enrichment term is represented by colored dots (red indicates high enrichment and blue indicates low enrichment)

To reflect a global overview of the regulatory functions of miRNAs, the GO terms of all target genes were analyzed through a GO annotation. Among the 2162 target genes, 1711 target genes had GO terms. As shown in Additional file 8: Figure S3, target prediction analysis showed that the identified targets regulated in a wide spectrum of biological processes, cellular components, and molecular functions. The target genes with molecular functions in nucleic acid binding presented the highest percentage, corresponding to 12.36%, followed by ATP binding, 10.7% (Additional file 8: Figure S3). GO enrichment analysis revealed that the top three terms were copper ion binding; oxidoreductase activity, oxidizing metal ions; oxidoreductase activity, acting on diphenols and related substances as donors (Fig. 3). Based on a KEGG pathway analysis, the target genes in this study were involved in 115 different pathways. The most top 5 enriched pathways include protein processing in endoplasmic reticulum, RNA transport, spliceosome, ubiquitin mediated proteolysis (Additional file 9: Figure S4). There were no enrichment results for KEGG analysis.

### Characterization of expressed miRNAs in berries

The relative expression levels of miRNAs were estimated as transcripts per million (TPM). The TPM values drastically varied among 9 miRNA samples. Some miRNAs were highly expressed in both ‘Fengzao’ and ‘Kyoho’, there are 36 sRNAs which accumulated at more than 1000 TPM (Additional file 10: Table S6).

**Fig. 4:**
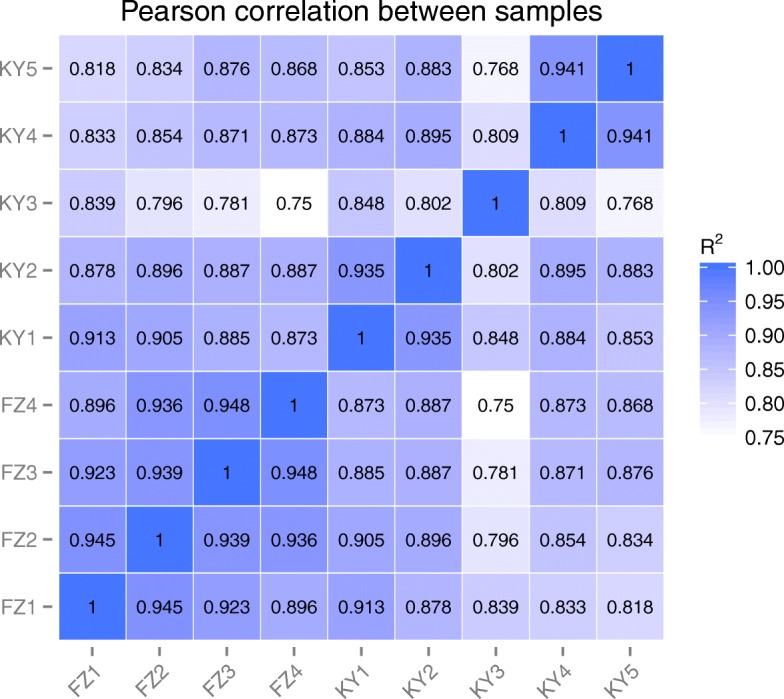
Correlation matrix of the 9 grape samples used to define the miRNA atlas. One minus Pearson correlation was used as a metric distance

In order to define the relationships of the 9 different samples from different berry stages of ‘Fengzao’ and ‘Kyoho’, a correlation matrix using Pearson coefficients (Fig. 4) were established to evaluate the level of similarity among the samples based on the miRNA expression levels. The high correlation was observed with average *R*^*2*^ ≥ 0.896 among ‘Fengzao’ samples (Fig. 4). Most of the correlation coefficients of ‘Kyoho’ samples was above 0.802 except the coefficients between KY3 and KY5 (Fig. 4).The correlation results showed that the berryies from different development periods of ‘Fengzao’ and ‘Kyoho’ are moderately related to each other, highlighting the similarities among these samples, except the KY3 samples.

**Fig. 5:**
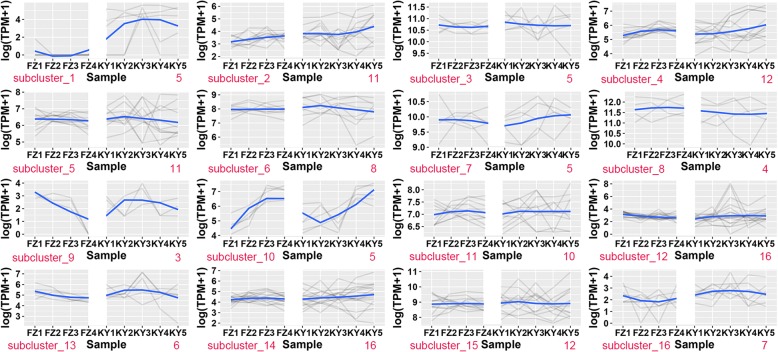
Cluster anlyses of the miRNAs expression patterns across various developmental stages in ‘Fengzao’ and ‘Kyoho’. Clustering was performed using k-means statistics. X-axis represents different sampling time points and Y-axis represents the expression level of miRNAs (log (TPM + 1)). The blue line was the fitted curve to match the expression change trend along the berry development

To better understand the overall expression profile of the common miRNAs in 9 samples, K-means clustering method were conducted further to elucidate the overall expression trends of the miRNAs based on the value of Log10(TPM + 1) –transformed. As a result, cluster analysis of the expression patterns divided these miRNAs into 16 groups (Fig. 5). The miRNAs and corresponding subclusters for the 16 groups are shown in Additional file 8: Table S8 and Fig. 5. As shown in Fig. 5, most subclusters showed similar expression patterns between ‘Kyoho’ and ‘Fengzao’. Some differences in miRNA expression patterns were revealed between ‘Kyoho’ and ‘Fengzao’ in subcluster_1, subcluster_9, subcluster_10 (Fig. 5).

The subcluster_1 comprised miR3626-5p, miR3626-3p, novel_144, novel_169, and novel_139; the expression levels of these miRNAs were very low in ‘Fengzao’, but gradually increased in ‘Kyoho’during berry developmental stages. These miRNAs were annotated as Serine/threonine- protein kinase, Sodium/calcium exchanger membrane region, phosphopantetheine-binding domain and SEP domain. The subcluster_9 composed of novel_117, novel_128 and miR3624-5p (WD40 repeat, FAD-binding), they gradually decreased in ‘Fengzao’along the berry development, but fluctuated in ‘Kyoho’. The subcluster_10 included miR398a (Thioredoxin-like fold), miR169b (Actin-related protein), novel_182, novel_64 and novel_46, they gradually increased in ‘Fengzao’, while they firstly decreased in KY2 of ‘Kyoho’, then slowly increased.

### Identification and different expression of miRNAs

The normalized expression levels of miRNAs were compared between the samples from ‘Kyoho’ and ‘Fengzao’ to identify differentially expressed miRNAs based on the criteria (|fold-change| > =2, q-value < 0.05). The comparisons were made between any two adjacent sampling time points within ‘Fengzao’ or ‘Kyoho’, respectivley.

There are few common differentially expressed miRNAs obtained from pairwise comparisons of the samples. Considering just for the samples of ‘Fengzao’, only one miRNA, miR159a, was differentially expressed between any two adjacent sampling time points, i.e., were differentially expressed in all stages of ‘Fengzao’. While for ‘Kyoho’, there are six miRNAs, miR159a, miR164a, miR2111-3p, miR3625-5p, miR390 and miR828a. These results indicated that a larger number of miRNAs were differentially expressed during all stages of berry development.

The overall comparison between ‘Kyoho’ and ‘Fengzao’ revealed that novel_144, vvi-miR3626-3p, vvi-miR3626-5p, vvi-miR399b, vvi-miR399e, vvi-miR477b-3p were differentially expressed (Table 3). The information about the expression and target genes of the differently expressed miRNAs are listed in Table 3. Some miRNAs showed genotype-specific expression patterns. For example, several miRNAs were expressed only in ‘Kyoho’, but completely didn’t express in any berry development stages of ‘Fengzao’, such as novel_144, vvi-miR3626-3p and vvi-miR3626-5p (Table 3), indicating that these miRNAs may have a specific role in berry development of grape. Some miRNAs were down-regulated in ‘Fengzao’ when compared with ‘Kyoho’; these miRNAs are miR399b and miR399e. The target genes of these miRNAs are annotated as NADH-ubiquinone oxidoreductase, Acyl carrier protein (ACP), Acyl carrier protein (ACP), Serine/threonine- protein kinase, SEP domain, inner membrane protein, Sodium/calciumoi exchanger membrane region, Phosphate permease, Ubiquitin-related domain, etc. Contrary to this, miR477b-3p was upregulated in ‘Fengzao’. The annotation of miR477b-3p target genes are Ubiquitin-related domain, Tetratricopeptide repeat, methyltransferase, LRR receptor-like serine threonine-protein kinase gso1-like, etc.

**Table 3:** Significantly differentially expressed miRNAs identified in ‘Fengzao’ compared with ‘Kyoho’

miRNA	FZ	KY	log2FoldChange	pval	padj	Target genes	Annotation from GO and interPro
novel_144	0	43.34	−4.0075	2.02E-14	1.12E-12		
vvi-miR3626-3p	0	29.95	−3.582	2.15E-11	7.94E-10	VIT_06s0061g00770 VIT_11s0016g05110 VIT_01s0244g00030 VIT_18s0076g00210 VIT_03s0088g00450 VIT_10s0116g00210 VIT_16s0050g01870 VIT_14s0083g01170 VIT_16s0050g01490 VIT_02s0087g00400 VIT_18s0001g06780 VIT_10s0116g00290 VIT_19s0014g00220 VIT_12s0028g00300 VIT_04s0023g00150 VIT_14s0066g01000 VIT_14s0171g00260	50s ribosomal protein l9; Ribosomal protein L9/RNase H1, N-terminal protein brittle- chloroplastic amyloplastic-like; Mitochondrial carrier domai Acyl carrier protein (ACP); Polyketide synthase, phosphopantetheine-binding domain embryogenesis-associated protein emb8; Alpha/Beta hydrolase fold fkbp12-rapamycin complex-associated protein; Phosphatidylinositol 3−/4-kinase, PIK-related kinase hypothetical protein; UBX||Ubiquitin-related domain||SEP domain CAAX amino terminal protease family protein; CAAX amino terminal protease mitochondrial carrier protein; Mitochondrial substrate/solute carrier NADH-ubiquinone oxidoreductase 13 kda-b subunit; ETC complex I subuni protein notum homolog; Alpha/Beta hydrolase fold||Pectinacetylesterase probable serine threonine-protein kinase at1g54610-like; Protein kinase, ATP binding site||Serine/threonine- plant UBX domain-containing protein 3; SEP domain big map kinase; Serine/threonine- protein kinase ||Mitogen-activated protein (MAP) kinase, ATP binding site glutamyl-tRNA amidotransferase subunit a-like; Tetratricopeptide repeat-containing domain inner membrane protein; Tetratricopeptide repeat-containing domain, embrane insertase OXA1/ALB3/YidC phosphate phosphoenolpyruvate translocator; Drug/metabolite transporter phosducin-like protein 3; Phosducin, thioredoxin-like domain
vvi-miR3626-5p	0	82.19	−4.7967	1.11E-21	1.23E-19	VIT_10s0116g00910 VIT_10s0003g03500 VIT_16s0050g02710 VIT_09s0018g01840 VIT_08s0007g04400 VIT_00s0179g00190	receptor protein; Serine/threonine-protein kinase, Protein kinase, ATP binding site katanin p60 ATPase-containing subunit a-like 2-like; AAA+ ATPase domain||ATPase, AAA-type, core probable receptor-like protein kinase at1g67000-like; Wall-associated receptor kinase galacturonan-binding domain vacuolar cation proton exchanger 2; Sodium/calcium exchanger membrane region dead-box atp-dependent RNA helicase 38-like; P-loop containing nucleoside triphosphate hydrolase||RNA helicase transcription factor jumonji domain-containing protein; JmjC domain
vvi-miR399b	38.95	159.83	−1.5213	0.001754	0.032445	VIT_08s0007g02840 VIT_06s0004g04430 VIT_13s0067g03280	LETM1-like protein; LETM1-like ubiquitin carrier protein; Ubiquitin-conjugating enzyme, active site high affinity inorganic phosphate transporter; General substrate transporter||Phosphate permease
vvi-miR399e	18.72	76.74	−1.761	5.11E-06	0.000142	VIT_13s0067g03280 VIT_08s0007g02840 VIT_13s0067g03280 VIT_06s0009g02380	high affinity inorganic phosphate transporter; General substrate transporter||Phosphate permease LETM1-like protein; LETM1-like high affinity inorganic phosphate transporter; General substrate transporter||Phosphate permease pseudouridine-5 -phosphate glycosidase; Pseudouridine-5′-phosphate glycosidase
vvi-miR477b-3p	780.94	283.54	1.2555	0.001439	0.031955	VIT_18s0001g01600 VIT_13s0084g00080 VIT_09s0002g03460 VIT_06s0004g08220 VIT_18s0001g03710 VIT_12s0055g00360 VIT_18s0041g01430 VIT_09s0002g02670 VIT_18s0122g00260 VIT_13s0158g00050 VIT_07s0104g00810 VIT_01s0010g03820 VIT_01s0010g00380 VIT_18s0001g05870	ATP binding; Putative S-adenosyl-L-methionine-dependent methyltransferase 65-kda microtubule-associated protein 5-like protein regulator; Microtubule-associated protein, MAP65 clavaminate synthase-like protein; Taurine catabolism dioxygenase TauD/TfdA 26 s protease regulatory subunit 6b homolog; P-loop containing nucleoside triphosphate hydrolase ||ATPase tmv resistance protein n-like; Leucine-rich repeat|P-loop containing nucleoside triphosphate hydrolase protein-protein interaction regulator family protein; Pinin/SDK/MemA protein tmv resistance protein n-like; Leucine-rich repeat, typical subtype RNA methyltransferase family protein; RNA methyltransferase TrmA superkiller protein 3-like protein; Tetratricopeptide repeat hypothetical protein; Peptidase S10, serine carboxypeptidase||Alpha/Beta hydrolase fold biotin lipoate a b protein ligase family protein; Biotin/lipoate A/B protein ligase||Octanoyltransferase LRR receptor-like serine threonine-protein kinase gso1-like; Protein kinase, ATP binding site LRR receptor-like serine threonine-protein kinase gso1-like; Leucine-rich repeat, Protein kinase, ATP binding site pentatricopeptide repeat-containing protein at2g13600-like; Tetratricopeptide-like helical

### qRT-PCR assay

**Fig. 6:**
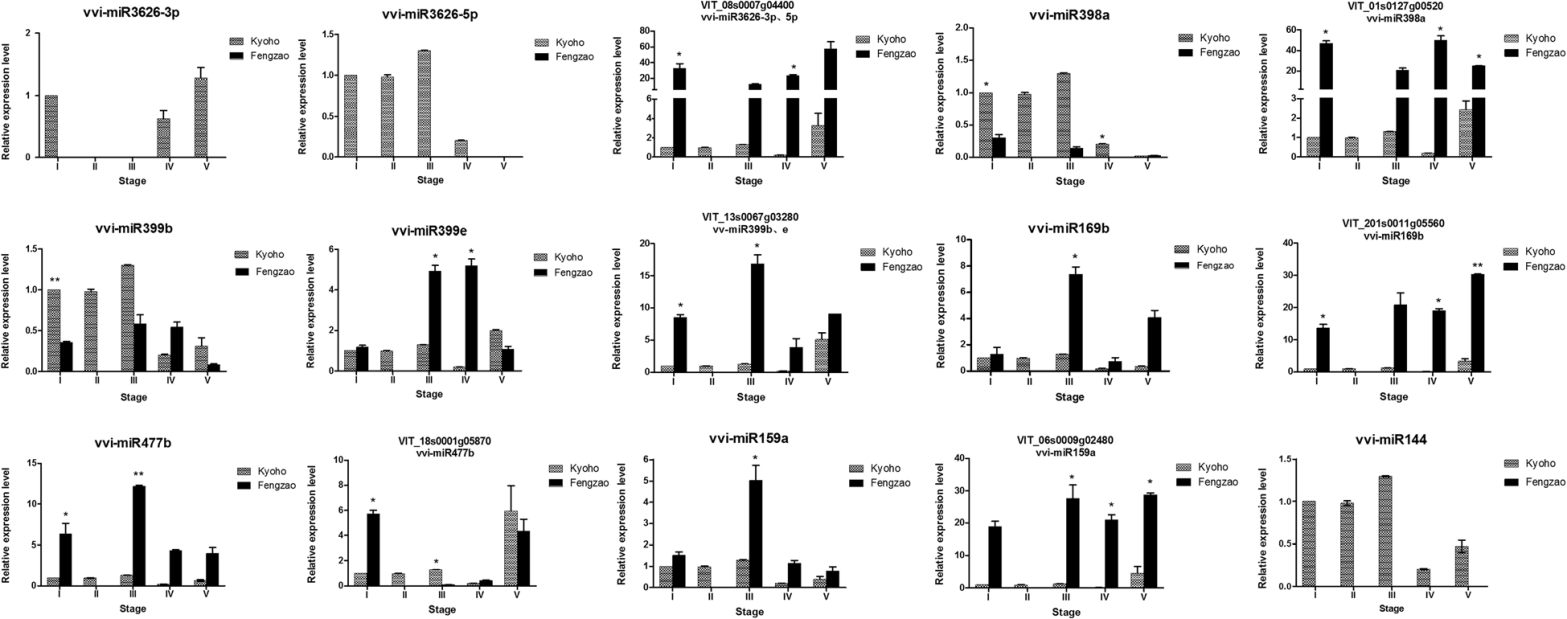
Stem-loop RT-qPCR validation of significantly differentially expressed miRNAs (vvi-) and Quantitative RT-PCR of their target genes (VIT_) at different berry development stages in ‘Fengzao’ and ‘Kyoho’. Relative quantity is based on the expression of the reference gene U6 and ubiquitin1 gene for miRNAs and target genes, respectively. X-axis indicates different stages (as indicated in Table 1) and Y-axis the expression of miRNA or target genes relative to reference gene. Data are mean ± SD from three biological replicates. *, *P* < 0.05; **, *P* < 0.01 by Student t test

To validate the transcriptome data, six differentially expressed and four specific miRNAs were selected for real-time quantitative reverse transcription (qRT)-PCR analysis. Most tested miRNAs showed similar expression trends in qRT-PCR data as sRNA-seq revealed (Fig. 6). The expression of novel_144, vvi-miR3626-3p, vvi-miR3626-5p miRNA indeed couldn’t be detected in ‘Fengzao’; accordingly, the expression of the target gene of vvi-miR3626-3p and vvi-miR3626-5p, VIT_08s0007g04400, expressed more in ‘Fengzao’ than in ‘Kyoho’. The expression of the target gene of miR399b and miR399e, VIT_13s0067g03280, also expressed more in ‘Fengzao’ than in ‘Kyoho’ due to the down-regulated expression of the miRNAs in ‘Fengzao’. The expression of the target genes of miR398 (VIT_01s0127g00520), miR169 (VIT_01s0011g05560) and miR 159 (VIT_06s0009g02480) expressed more in ‘Fengzao’ than in ‘Kyoho’. This result illustrated that our high-throughput data were reliable.

## Discussion

### Characterization of miRNAs during berry development

A lot of miRNAs have been identified and their expression atlas were established in grape [21–23, 27]. However, the miRNAs involved in grapes berry ripening process remained largely unknown. To characterize the miRNAs during grape berry development, nine libraries were constructed to identify miRNAs and profile their expressions across the key developmental stages of ‘Kyoho’ and ‘Fengzao’, respectively. As shown in Fig. 1, 21- to 24-nt sRNAs were dominant and occupied more than 80% of the total sRNAs. Especially, 21-nt sRNAs were more abundant than 24-nt sRNAs (Fig. 1), which was consistent with the previous studies in grape [22, 23, 27]. It showed that the expression levels of miRNAs varied at different developmental stages, indicating miRNAs play diverse roles during grape berry development.

Sequence analysis of sRNAs resulted in characterization of 108 known miRNAs and 61 novel miRNAs (Additional file 2: Table S2). The known miRNAs belonged to 35 conserved miRNA families and most of them were highly conserved among diverse plant species (Additional file 5, Figure S1). Normally, conserved plant miRNAs regulate homologous target genes at identical target sites and that these miRNAs may play the similar function in different species [28]. Target gene analysis showed that a single miRNA simultaneously has multiple target genes (Additional file 4: Table S4), such as miR156, miR159, miR172, and miR164, which is consistent with the previous reports [22]. Meanwhile, some miRNAs from different families could have the same target genes. The similar cases were documented in *Lycium barbarum* [18], tomato [29] and melon [12].

This study revealed many commonly expressed miRNAs as the previous researches [22, 23] despite the employments of very different varieties and berry stages, especially for the most highly expressed miRNAs such as miR156, miR159, miR172 and miR164. The amount of identified known and novel miRNAs were a little more than that of Wu et al. [3]. They analyzed small RNA expression profiles in navel orange of ‘Fengjie 72–1’ and its spontaneous late-ripening mutant ‘Fengwan’ at 170 days after flowering (DAF). They identified 107 conserved miRNAs and 21 novel miRNAs. It may be due to more fruit developmental stages covered in this study.

In contrast to conserved miRNAs, novel miRNAs are usually species-specific, which expressed at low levels and appeared to lack targets based on the current criteria or the research limits. In this study, only one novel miRNA (novel_141) was successfully predicted to target protein- coding genes, suggesting that most novel miRNAs have as yet undetermined roles at present.

### Specific expressed miRNAs in ‘Kyoho’

The overall comparison of miRNAs expressions showed that all the miRNAs expressed in ‘Fengzao’ also expressed in ‘Kyoho’, but 10 miRNAs (novel_139, novel_144, vvi-miR156a, vvi-miR156e, vvi-miR171h, vvi-miR3626-3p, vvi-miR3626-5p, vvi-miR845a, vvi-miR845c, vvi-miR845d) only presented in ‘Kyoho’. It suggested that the mutation in ‘Fengzao’ had resulted in the alteration of the corresponding miRNA expressions. MicroRNA regulates gene expression through inhibiting translation or degrading mRNA at a certain site, i.e., it down-regulates the expression of its target gene [30]. This implies that expression of miRNAs and their targets were negatively correlated. So, the miRNAs only expressed in ‘Kyoho’ means that the target of these miRNAs in ‘Fengzao’ were accordingly not suppressed and up-regulated. Gao et al. [1] demonstrated the up-regulation of the target genes during the ripening of wild-type tomato fruit, while their concurrent down-regulation in never-ripe *rin* mutant. Palumbo et al. [31] also observed that some miRNAs are deactivated and the switch genes are expressed during the transition from immature to mature growth of grape berry. Interestingly, vvi-miR156e, vvi-miR3626-5p, vvi-miR845a and vvi-miR845d were as parts of some miRNAs identified by Palumbo et al. [31] as miRNA/switch gene pairs for the transition from immature-to-mature stage in grape, only expressed in ‘Kyoho’ in this study. Pilati et al. [32] also showed that 13 out of 80 candidate “ABA-responsive switch genes” were predicted to be regulated post-transcriptionally by miRNAs including vi-miR156e, vvi-miR3626-5p and vvi-miR845a. These evidences suggested the important roles of these miRNAs for the switching of grape berry development. It need to be further explored in the future.

Kullan et al. [22] showed that miR156 and miR164 involving in grape berry ripening. miR156 decreased from the vegetative to the reproductive phase of *Arabidopsis* and its targets, *SQUAMOSA PROMOTER BINDING PROTEIN-LIKE* (SPLs), are key participators in regulating plant phase transitions [33]. The target gene of miR164, Vv-NAC, has been demonstrated a special function for grape berry ripening [34]. Several miR156-targeted genes were differentially expressed in pre- and post-anthesis ovaries of tomato, indicating its regulation role for fruit development [35]. MiR156/157 could affect the tomato ripening process by modulating the known ripening regulators *CNR* and *SIAP2a* [36]. vvi-miR 156a and vvi-miR156e only expressed in ‘Kyoho’ as confirmed by qRT-PCR (data not shown), which suggested that they may be related to the early-ripening mutation of ‘Fengzao’ due to its absence in ‘Fengzao’.

Specific set of miRNAs of different *Vitis* varieties and species have been revealed [22]. For example, Kullan et al. [22] and Mica et al. [21] previously didn’t detect the expression of vvi-miR845 in grape. But the expression of vvi-miR845a, vvi-miR845c, vvi-miR845d were detected in ‘Kyoho’ in this study. Palumbo et al. [31] showed that vvi-miR845a and vvi-miR156e had the same target gene, Pyruvate dehydrogenase kinase (VIT_214s0060g00420). Downregulation of the pyruvate dehydrogenase gene PDHE1α by VIGS had inhibited respiration and ATP biosynthesis, whilst promoted the accumulation of sugar, ABA and ETH, which then accelerated the ripening of strawberry [37]. And both of them were absent in ‘Fengzao’, indicated that vvi-miR845 and vvi-miR156 may have the similar roles for the grape berry ripening as in strawberry. In this study, vvi-miR845a, vvi-miR845c and vvi-miR845d were all predicted to target Gag-polypeptide of LTR *copia*-type (VIT_209s0002g08320) retrotransposon. Previous studies have suggested retrotransposon was closely related to the formation of grape bud mutant [38]. It need to be further explored whether the early-ripening mutant of ‘Fengzao’ is related to retrotransposon or not.

### Expression patterns of miRNAs between ‘Fengzao’ and ‘Kyoho’

Expression profiles based on the k-means method showed most of the miRNAs had the similar expression patterns between ‘Fengzao’ and ‘Kyoho’ during fruit development (Fig. 5), which indicated that the mutation in ‘Fengzao’ didn’t alter the expressions of miRNAs very much compared to ‘Kyoho’. The results also suggested that miRNAs from the same cluster have parallel expression patterns (Fig. 5). Furthermore, different clusters contained some similar miRNAs (from the same miRNA family) (Fig. 5). This implied that those miRNAs even from the same family had different expression patterns and they synergistically regulate the berry development. A similar situation has been also reported [17].

The differential expression profiles of some miRNAs were identified at different berry developmental stages between ‘Fengzao’ and ‘Kyoho’ during berry development (Fig. 5). For example, 5 miRNAs in subcluster 1 of Fig. 5, including miR3626-5p, miR3626-3p, novel_144, novel_169, and novel_139, their expression pattern were entirely different between ‘Kyoho’ and ‘Fengzao’. They expressed very lowly in ‘Fengzao’ and gradually increased in ‘Kyoho’ along the berry development. The expression profiles of miRNAs in subcluster 10 of Fig. 5, miR398a, miR169b, novel_182, novel_46 and novel_64 are also different between ‘Fengzao’ and ‘Kyoho’. The difference in expression profiles may suggest that the mutations in ‘Fengzao’ modified the miRNAs expression during berry development.

The main targets of miR398 is copper/zinc superoxide dismutase (Cu/Zn-SOD, CSD), a scavenger enzyme of ROS (reactive oxygen species), which is related to the oxidative stress [39, 40]. The overexpression of miR398 had led to the downregulation of CSD1 and CSD 2 enzymes in rice [41]. In our previous study [8], RNAseq analysis revealed that SOD was one of the most significantly differently expressed genes between ‘Fengzao’ and ‘Kyoho’ during berry development and the overall expression level of SOD in ‘Fengzao’ was lower than that in ‘Kyoho’, except at the stage of veraison. The expression of miR398 increased more in ‘Fengzao’ than in ‘Kyoho’ (subcluster 10 of Fig. 5) which also verified our previous results [8]. And the activity of SOD enzyme in ‘Fengzao’ indeed was lower than that in ‘Kyoho’ except at the stage of veraison [10].

The target genes of miR169 were Jasmonate ZIM Domain (JAZ) and nuclear transcription factor Y subunit A-3 (NFYA-3) in tomato [20, 41]. Zeng et al. [18] suggested that miR169 involved in the fruit development of *Lycium barbarum*, it expressed at a higher level in fruit than in flowers and leaves [42]. Ripening can be considered as a stressful process with a progressive increase in oxidation [43, 44]. Overexpression of miR169 caused significantly down-regulation of its target genes and induced the increased drought tolerance of tomato [45]. Accompanied the gradually increasing expression of miR169 in ‘Fengzao’, and the overall oxidation status (ROS levels) in ‘Fengzao’ was indeed higher than that in ‘Kyoho’ [10] .

### Differentially expressed miRNAs between ‘Fengzao’ and ‘Kyoho’

Only miR159a was significantly differentially expressed between any adjacent stages during the berry development either in ‘Fengzao’ or ‘Kyoho’, but it was not significantly differentially expressed between ‘Fengzao’ and ‘Kyoho’, which suggested that miR159a constitutively expressed along the grape berry development. Based on the expression analysis of miRNAs, the significantly differentially expressed miRNAs between ‘Fengzao’ and ‘Kyoho’ were novel_144, vvi-miR3626-3p, vvi-miR3626-5p, vvi-miR399b, vvi-miR399e and vvi-miR477b-3p. Among these miRNAs, novel_144, vvi-miR3626-3p, vvi-miR3626-5p only expressed in ‘Kyoho’ and their expression patterns were entirely distinct between ‘Fengzao’ and ‘Kyoho’ (Fig. 5: subcluster1). vvi-miR3626 was previously not detected in grape [21, 22].

It is known that miR159 target the GAMYB transcription factors to affect flowering time [46]. The miR159­GAMYB system is conserved for the vegetative­to­reproductive phase transition in plants [40]. Grape targets of miR159 include the GAMYB transcription factors MYB33, MYB65, and MYB101 [27], which participate in the signaling process induced by ABA accumulation in the presence of stress [47].The up-regulation of miR159 could inhibit ABA signaling through down-regulation of MYB transcription factors [47]. Abscisic acid has key role for the onset of grape berry ripening [48]. Therefore, it could be comprehensible for the differentially expressed of miR159 during the berry development both in ‘Fengzao’ and ‘Kyoho’. Similarly, miR159b and miR319a/b/c/d had high expression level at early development stages, and were down-regulated at the late stages of fruit ripening in melon [12]. Fa-MIR159a transcript reached its highest expression level during the green stage and subsequently decreased significantly during the white and red stages strawberry [49].

The targets of vvi-miR3626-3p, vvi-miR3626-5p in this study are predicted as DEAD-box ATP-dependent RNA helicase, fkbp12-rapamycin complex-associated protein, Serine/threonine- protein kinase and phosphate phosphoenolpyruvate translocator, etc. (Table 3). The DEAD-box RNA helicases are the largest family of RNA helicases, and DEAD-box helicase is believed to play crucial roles in plant growth and development [50]. Previous studies have shown that the overexpression of helicase genes could elevate SOD activity [51, 52]. In this study, the lacking expression of vvi-miR3626-3p and vvi-miR3626-5p in ‘Fengzao’ suggested the SOD activity should decrease accordingly. Interestingly, our previous results indeed confirmed this [8, 10]. SOD is an important ROS scavenging enzyme integral to plant stress tolerance [53]. Two DEAD-box helicases function in abscisic acid (ABA)-dependent and ABA-independent abiotic stress signaling pathways in *Arabidopsis thaliana* [54]. Abscisic acid is a major regulator of grape berry ripening [48] and ABA also involves in plant adaptive responses to abiotic and biotic stresses processes [55]. The functional involvement of a putative helicase in the antioxidative responses in alfalfa has also been reported [52]. Wu et al. [3] proposed that stress response process may play an important role during citrus fruit ripening when the comparative analyses of miRNAs expression between a spontaneous late-ripening sweet orange mutant and its wild-type were conducted using small RNA sequencing. Pilati et al. [56] demonstrated an oxidative burst in ‘Pinot Noir’ at veraison stage and ROS could participate to the regulatory network of fruit development in grape [57].

Therefore, taken the above mentioned expression patterns of vvi-miR398, vvi-miR169b and vvi-miR159a; the differentially expression of vvi-miR3626-3p and vvi-miR3626-5p; and our previous results [8, 10] into consideration, we deduced that the stress response (ROS related) play a significant role during grape berry ripening and partially accounted for the early-ripening mutant of ‘Fengzao’. Furhtermore, H_2_O_2_ as the exogenous ROS stress indeed promoted the early-ripening of ‘Kyoho’ [10].

miR399 involved in plant responses to phosphate starvation by targeting an ubiquitin conjugating enzyme (UBC) gene associated with the function of MYB transcription factor [58]. UBC24, functions as a repressor that prevents excessive accumulation of Pi [59]. In this study, vvi-miR399b and vvi-miR399e targeted to high affinity inorganic phosphate transporter. Phosphorus (Pi) is an essential nutrient for optimal plant growth and development. The presence of miR399 in response to phosphorus deficiency has been confirmed in various plants [58]. miR399 family members were also differentially expressed in different grape genotypes, which showed they were connected to grape berry development [23]. Okamoto et al. [60] showed that phosphate enhanced ROS production. Jia et al. [61] proposed that overexpression of AtMPT3 elevated the Pi concentration in mitochondrial matrix, which accelerated the subsequent processes of electron transport, ATP biosynthesis, ROS accumulation and PCD. In this study, both vvi-miR399b and vvi-miR399e were down-regulated in ‘Fengzao’, which should theoretically result in the elevation of Pi content which may further facilitate the ROS production to promote the early-ripening of ‘Fengzao’ based on the above assumptions.

miR477 was found to target GRAS family transcription factor in grape [21, 22]. GRAS involved in many processes of plant growth and development, as well as in plant disease resistance and abiotic stress responses [62]. Grimplet et al. [62] revealed the possible functions of GRAS genes in grape development and stress responses. Several grape GRAS genes showed differential expression among different berry ripening stages [63]. In this study, vvi-miR477 was upregulated in ‘Fengzao’, which suggested its connection with the early-ripening of ‘Fengzao’.

Candidate miRNAs that showed remarkably differential expression levels were further confirmed by Quantitative RT-PCR (qRT-PCR). Totally, 9 known- and 1 novel- miRNAs were chosen for qRT-PCR confirmation; most of the results are consistent with the sequencing data (Fig. 6).

### A possible network may contribute to the regulation of early-ripening of ‘Fengzao’

**Fig. 7:**
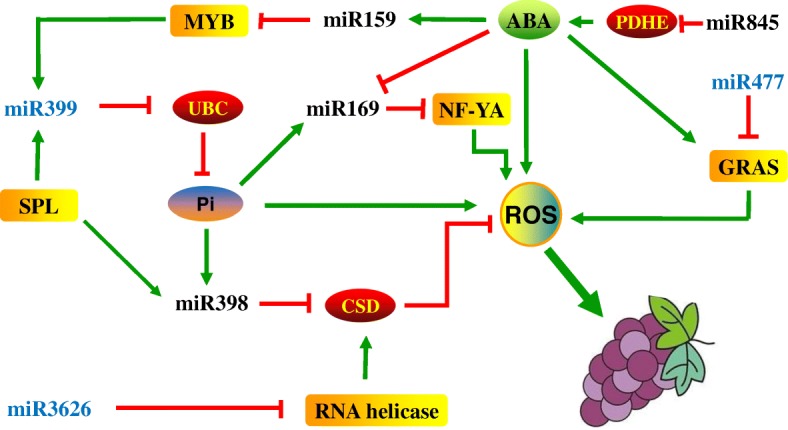
The speculated possible regulatory network of miRNAs-TFs-Genes in early-ripening of ‘Fengzao’. PDHE: pyruvate dehydrogenase, CSD: superoxide dismutase, ROS: reactive oxygen species, NFYA: nuclear transcription factor Y subunit A, PHO2: PHOSPHATE2, SPL: squamosa-promoter binding protein-like

Based on the expression profiles and differentially expressed miRNAs between ‘Fengzao’ and ‘Kyoho’, miR159-MYBs, miR169-NF-Y subunit, miR398-CSD, miR3626- RNA helicase, miR399-phosphate transporter, and miR477-GRAS transcription factor were integrated to further explore their potential roles for early-ripening of ‘Fengzao’ (Fig. 7).

miR159 acts as a negative regulator, while AtMYB33 and AtMYB101 act as positive regulators, of the ABA response [47]. MYB transcription factor was able to activate miR399, which responded to phosphate (Pi) starvation in *Arabidopsis* [58, 64]. Upon Pi deprivation, the expression of miR156, miR399, miR778, miR827, and miR2111 were induced, whereas the expression of miR169, miR395, and miR398 was repressed [65]. During Pi starvation, the upregulated miR399 regulates Pi homeostasis by suppressing the expression of UBC gene [65].

In *Arabidopsis*, SPL3 could directly activate the expression of several Pi starvation inducible genes. A miR156-SPL3 module involved in negative feedback regulation of miR399-mediated pathways in response to low P was proposed [66]. SPL7 is essential for the expression of miR397, miR398, miR408, miR857 [67]. miR169 which targets multiple members of subunit A of the NF-Y transcription factor complex, was downregulated by N or P starvation [68] and was also downregulated by ABA [69]. The PAT1 branch of GRAS family was markedly induced by ABA [70]. NtGRAS1 from tobacco was strongly induced by various stimulants that raise the intracellular reactive oxygen (ROS) levels [71].

Based on the above progress and profiles of miRNAs detected in this study, we presumably constructed the network of miRNAs which regulating early-ripening of ‘Fengzao’ as Fig. 7 showed. The final connecting point of these miRNAs could be concentrated on the regulation of ROS level. We have shown the effect of ROS for accelerating the early-ripening of ‘Kyoho’ [10], which strongly support the conclusions of this study.

These results indicated that the regulation network of miRNAs for fruit ripening are quite complicated, and elucidation of the molecular mechanisms underlying the interplay between miRNA and their target genes involved in early-ripening of grape requires further study.

## Conclusions

In the present study, we performed a comparative analysis of miRNA expression profiles between ‘Fengzao’ and ‘Kyoho’ along grape berry development. Our results revealed that specific miRNAs were differentially regulated during berry development. The expression patterns of several key miRNAs and their target genes during grape berry development and ripening stages was examined. According to the expression analysis and previous reports, miR169-NF-Y subunit, miR398-CSD, miR3626- RNA helicase, miR399-phosphate transporter and miR477-GRAS transcription factor were selected as the candidates for further investigations of miRNA regulation of early-ripening of ‘Fengzao’. The results of this study contribute to the understanding of the role of miRNAs in grape berry early-ripening and will provide new sights for small RNA engineering approach to grape molecular breeding.

## Methods

### Plant materials

The berries of ‘Fengzao’ and ‘Kyoho’ at different stages were collected in 2016 from the farm of Henan University of Science & Technology, Luoyang, China. Berries from 5 individual vines were pooled and immediately frozen in liquid nitrogen, then stored at − 80 °C until further use. Based on the comprehensive considerations of the grape growth stages which Coombe [72] established and our previous results [6–9], the berries at periods of E-L 33 (hard green berries), E-L 34 (starting to soften), E-L 35 (véraison), E-L 37 (sugar and anthocyanins accumulation, and active growth, Berries not quite ripe) were sampled. Because the development status of the berry was largely different between ‘Fengzao’ and ‘Kyoho’, especially for the interval between E-L33 and E-L34 in ‘Kyoho’, the sampling points in ‘Kyoho’ are one more than ‘Fengzao’. The specific sampling time and corresponding development stages are shown in Table 1.

### Small RNA library construction and sequencing

Total RNA was extracted from the berry according to the method of Rienth et al. [73]. The RNA purity and integrity were evaluated using Agilent Bioanalyzer 2100 system (Agilent Technologies, CA, USA) and quantified by Qubit® 2.0 Flurometer (Life Technologies, CA, USA). A total amount of 3 μg total RNA per sample were used as input material for the small RNA library construction. Sequencing libraries were generated using NEBNext® Multiplex Small RNA Library Prep Set for Illumina®(NEB, USA.) following manufacturer’s recommendations, then sequencing on an Illumina Hiseq 2500/2000 platform at Novogene Corporation (China).

### Bioinformatics analysis of the sequencing data

The raw reads were first cleaned up and filtered by removing adapter sequences and low-quality reads containing ploy-N, with adapter contaminants or the insert tag, and lengths < 18 nt. High-quality clean small RNA tags were aligned to grape reference sequence (ftp://ftp.ensemblgenomes.org/pub/plants/release-23/fasta/vitis_vinifera) with Bowtie 0.12.9. Then, the clean sequences were annotated by searching against RepeatMasker and Rfam 11.0 databases to determine repeat, rRNA, tRNA, snRNA and snoRNA. The remaining sequences were mapped to miRBase 20.0 to identify known miRNAs. Both miREvo [74] and mirdeep2 [75] were employed to predict novel miRNA through exploring the secondary hairpin structure, the Dicer cleavage site and the minimum free energy of the small RNA tags unannotated in the former steps. At the same time, custom scripts were used to obtain the identified miRNA counts as well as base bias on the first position with certain length and on each position of all identified miRNA respectively. The miRNA editing sites located on the known miRNA sequences were analyzed by aligning all the sRNA tags to mature miRNA, allowing one mismatch.

### miRNA expression and comparison between ‘Kyoho’ and ‘Fengzao’

miRNA expression levels were normalized by TPM (transcript per million) with the criteria: Normalized expression = mapped read count/ Total reads*1000000. DESeq package 1.12.0. was used for differential expression analysis of miRNAs between ‘Kyoho’ and ‘Fengzao’ at the whole level including all the berry development stages. The *P*-values were adjusted using the Benjamini & Hochberg method. Corrected P-value of 0.05 and |log_2_(foldchange) > 1| were set as the threshold for significantly differential expression. The expression change patterns of individual miRNAs were characterized by K-means clustering in both ‘Kyoho’ and ‘Fengzao’ based on log 10 (1 + TPM) normalization. All statistical analyses were performed in R version 3.4.3.

### miRNA-target prediction and enrichment analysis

Predicting the target genes of miRNA was performed in psRobot software [76]. The target gene candidates of differentially expressed miRNAs were used for Gene Ontology (GO) enrichment analysis. ClusterProfiler 3.8.0 was implemented for GO and Kyoto Encyclopedia of Genes and Genomes (KEGG) pathway enrichment analysis [77]. GO terms or pathways that had a *p* value less than 0.05 after Bonferroni correction were scored as significant.

### Validation of miRNA expression with qRT-PCR

To validate the expression of the differentially expressed miRNAs and their role in grape berry development, differently expressed miRNAs were chosen to design miRNA-specific stem-loop primers for stem-loop qPCR analysis. The miRNA specific forward primers and stem-loop RT primers were designed with the primer premier 5.0 software. All primers sequences were provided in Additional file 11: Table S7. Stem-loop qRT-PCR was performed to validate the expressions of miRNAs with three biological replicates based on a previous method [3]. The samples were newly collected from three individual vines in 2017; each was a biological replicate. cDNAs were reverse transcribed from total RNAs using SuperScript III Reverse Transcriptase (Invitrogen, USA). U6 and ubiquitin1 gene were used as the internal control for qRT-PCR of miRNAs and target genes. For each reaction, 0.5 μL cDNA and 5 μL SYBR Green PCR Master mix (Toyobo, Osaka, Japan), and 0.5 μM forward and reverse primers were mixed. PCR was run in triplicate at 95 °C for 1 min, followed by 40 cycles at 95 °C for 15 s and 60 °C for 1 min with CFX96 Real Time PCR Detection System (Bio-Rad, USA). Meanwhile, the expression profiles of nine predicted target genes were also tested by qRT-PCR with primers listed in Additional file 6. The relative expression changes of mature miRNAs and predicted target genes were calculated using the 2^-ΔΔCt^ method.

## Additional files

Additional file 1: **Table S1.** The summary of the sequenced reads of different samples. (XLSX 9 kb)

Additional file 2: **Table S2.** The composition of each miRNA family identified in this study. (XLSX 12 kb)

Additional file 3: **Table S3.** The mature sequenced of all known and novel miRNAs. (XLSX 14 kb)

Additional file 4: **Table S4.** The identified miRNAs in ‘Fengzao’ and ‘Kyoho’, respectively. (XLSX 115 kb)

Additional file 5: **Figure S1.** Deeply sequence conserved and previously reported miRNA families detected from developing berries of ‘Fengzao’ and ‘Kyoho’. miRNA families (columns) are conserved between plants families (rows) for plant species represented in miRBase release 21. The bar represents the scale of the numbers of the miRNAs after the Z-score standardization. (PDF 690 kb)

Additional file 6: **Table S5.** Target gene prediction results of all the miRNAs identified in this study. (XLSX 10 kb)

Additional file 7: **Figure S2.** miRNA variants and their nucleotide bias position. A: MiRNA nucleotide bias at each miRNA position. B: First nucleotide bias for the first position of 18- to 30-nt miRNAs. Relative nucleotide bias at each miRNA position compared with the total RNA. (PDF 7480 kb)

Additional file 8: **Figure S3.** GO categories and distribution of miRNA targets gene of all the miRNAs identified in this study. The left-hand-side scale is the percent of the targeted gene numbers corresponding to the GO terms. (PDF 2795 kb)

Additional file 9: **Figure S4.** KEGG analysis of the 20 most enriched pathways. The coloring of the q-values indicates the significance of the rich factor. The circle indicates the target genes that are involved, and the size is proportional to the gene numbers. The x-axis represents name of enrichment pathway. The Y-axis represents rich factor. (PDF 6 kb)

Additional file 10: **Table S6.** The expression data (TPM value) of known and novel miRNAs and the location of clusters based on the K-means method in this study. (XLSX 42 kb)

Additional file 11: **Table S7.** The primers of miRNAs and genes used for qRT-PCR verification. (XLS 10 kb)

## Abbreviations

CSD: Copper/zinc superoxide dismutase
GRAS: After the first three members: Gibberellic-acid insensitive (gai), Repressor of gai (rga) and Scarecrow (scr)
miRNA: MicroRNA
NF-YA: Nuclear Factor Y A subunits
PDHE: Pyruvate dehydrogenase
PHO2: Phosphate2
ROS: Reactive oxygen species
SPLs: *Squamosa promoter binding protein-like*
sRNA: Small RNA
TFs: Transcription factors
TPM: Transcripts per million
UBC: Ubiquitin-conjugating conserved domain

## References

[1] Gao C, Ju Z, Cao D, Zhai B, Qin G, Zhu H, Fu D, Luo Y, Zhu B (2015). MicroRNA profiling analysis throughout tomato fruit development and ripening reveals potential regulatory role of *RIN* on microRNAs accumulation. Plant Biotechnol J.

[2] Giovannoni J, Nguyen C, Ampofo B, Zhong S, Fei Z (2017). The epigenome and transcriptional dynamics of fruit ripening. Annu Rev Plant Biol.

[3] Wu J, Zheng S, Feng G, Yi H (2016). Comparative analysis of miRNAs and their target transcripts between a spontaneous late-ripening sweet orange mutant and its wild-type using small rna and degradome sequencing. Front Plant Sci.

[4] Reuscher S, Isuzugawa K, Kawachi M, Oikawa A, Shiratake K (2014). Comprehensive elemental analysis of fruit flesh from European pear ‘La France’ and its giant fruit bud mutant indicates specific roles for B and Ca in fruit development. Sci Hortic.

[5] Kumar V, Irfan M, Ghosh S, Chakraborty N, Chakraborty S, Datta A (2016). Fruit ripening mutants reveal cell metabolism and redox state during ripening. Protoplasma.

[6] Guo DL, Zhang GH (2015). A new early-ripening grape cultivar – ‘Fengzao. Acta Hortic.

[7] Guo DL, Guo MX, Zhang GH (2014). Comparisons of berry development characteristics between the early ripening bud mutants of grape and their parents. Plant Physiol J.

[8] Guo DL, Xi FF, Yu YH, Zhang XY, Zhang GH, Zhong GY (2016). Comparative RNA-Seq profiling of berry development between table grape ‘Kyoho’and its early-ripening mutant ‘Fengzao’. BMC Genomics.

[9] Guo DL, Yu YH, Xi FF, Shi YY, Zhang GH (2016). Histological and molecular characterization of grape early ripening bud mutant. Int J Genomics.

[10] Xi F-F, Guo L-L, Yu Y-H, Wang Y, Li Q, Zhao H-L, Zhang G-H, Guo D-L (2017). Comparison of reactive oxygen species metabolism during grape berry development between ‘Kyoho’and its early ripening bud mutant ‘Fengzao’. Plant Physiol Biochem.

[11] Bartel DP (2004). MicroRNAs: genomics, biogenesis, mechanism, and function. Cell.

[12] Zhang H, Yin L, Wang H, Wang G, Ma X, Li M, Wu H, Fu Q, Zhang Y, Yi H (2017). Genome-wide identification of Hami melon miRNAs with putative roles during fruit development. PLoS One.

[13] Zhang X, Wang W, Wang M, Zhang HY, Liu JH (2016). The miR396b of *Poncirus trifoliata* functions in cold tolerance by regulating acc oxidase gene expression and modulating ethylene-polyamine homeostasis. Plant Cell Physiol.

[14] Liu Y, Wang L, Chen D, Wu X, Huang D, Chen L, Li L, Deng X, Xu Q (2014). Genome-wide comparison of microRNAs and their targeted transcripts among leaf, flower and fruit of sweet orange. BMC Genomics.

[15] Saminathan T, Bodunrin A, Singh NV, Devarajan R, Nimmakayala P, Jeff M, Aradhya M, Reddy UK (2016). Genome-wide identification of microRNAs in pomegranate (*Punica granatum* L.) by high-throughput sequencing. BMC Plant Biol.

[16] Fang Y-N, Zheng B-B, Wang L, Yang W, Wu X-M, Xu Q, Guo W-W (2016). High-throughput sequencing and degradome analysis reveal altered expression of miRNAs and their targets in a male-sterile cybrid pummelo (*Citrus grandis*). BMC Genomics.

[17] Wu J, Wang D, Liu Y, Wang L, Qiao X, Zhang S (2014). Identification of miRNAs involved in pear fruit development and quality. BMC Genomics.

[18] Zeng S, Liu Y, Pan L, Hayward A, Wang Y (2015). Identification and characterization of miRNAs in ripening fruit of *Lycium barbarum* L. using high-throughput sequencing. Front Plant Sci.

[19] Jose Ripoll J, Bailey LJ, Mai QA, Wu SL, Hon CT, Chapman EJ, Ditta GS, Estelle M, Yanofsky MF (2015). microRNA regulation of fruit growth. Nat Plants.

[20] Karlova R, van Haarst JC, Maliepaard C, van de Geest H, Bovy AG, Lammers M, Angenent GC, de Maagd RA (2013). Identification of microRNA targets in tomato fruit development using high-throughput sequencing and degradome analysis. J Exp Bot.

[21] Mica E, Piccolo V, Delledonne M, Ferrarini A, Pezzotti M, Casati C, Del Fabbro C, Valle G, Policriti A, Morgante M (2009). High throughput approaches reveal splicing of primary microRNA transcripts and tissue specific expression of mature microRNAs in *Vitis vinifera*. BMC Genomics.

[22] Kullan JB, Pinto DLP, Bertolini E, Fasoli M, Zenoni S, Tornielli GB, Pezzotti M, Meyers BC, Farina L, Pè ME (2015). miRVine: a microRNA expression atlas of grapevine based on small RNA sequencing. BMC Genomics.

[23] Paim Pinto DL, Brancadoro L, Dal Santo S, De Lorenzis G, Pezzotti M, Meyers BC, Pe ME, Mica E (2016). The influence of genotype and environment on small RNA profiles in grapevine berry. Front Plant Sci.

[24] Naoumkina M, Thyssen GN, Fang DD, Hinchliffe DJ, Florane CB, Jenkins JN (2016). Small RNA sequencing and degradome analysis of developing fibers of short fiber mutants Ligon-lintles-1 (li 1 ) and −2 (li 2 ) revealed a role for miRNAs and their targets in cotton fiber elongation. BMC Genomics.

[25] Omidvar V, Mohorianu I, Dalmay T, Fellner M (2015). Identification of miRNAs with potential roles in regulation of anther development and male-sterility in 7B-1 male-sterile tomato mutant. BMC Genomics.

[26] Song C, Zhang D, Zheng L, Zhang J, Zhang B, Luo W, Li Y, Li G, Ma J, Han M (2017). miRNA and degradome sequencing reveal mirna and their target genes that may mediate shoot growth in spur type mutant “Yanfu 6”. Front Plant Sci.

[27] Pantaleo V, Szittya G, Moxon S, Miozzi L, Moulton V, Dalmay T, Burgyan J (2010). Identification of grapevine microRNAs and their targets using high-throughput sequencing and degradome analysis. Plant J.

[28] de Fátima Rosas-Cárdenas F, de Folter S (2017). Conservation, divergence, and abundance of MiRNAs and their effect in plants: Springer International Publishing AG.

[29] Liu M, Yu H, Zhao G, Huang Q, Lu Y, Ouyang B (2017). Profiling of drought-responsive microRNA and mRNA in tomato using high-throughput sequencing. BMC Genomics.

[30] Chien P-S, Chiang C-B, Wang Z, Chiou T-J (2017). MicroRNA-mediated signaling and regulation of nutrient transport and utilization. Curr Opin Plant Biol.

[31] Palumbo MC, Zenoni S, Fasoli M, Massonnet M, Farina L, Castiglione F, Pezzotti M, Paci P (2014). Integrated network analysis identifies fight-club nodes as a class of hubs encompassing key putative switch genes that induce major transcriptome reprogramming during grapevine development. Plant Cell.

[32] Pilati S, Bagagli G, Sonego P, Moretto M, Brazzale D, Castorina G, Simoni L, Tonelli C, Guella G, Engelen K (2017). Abscisic acid is a major regulator of grape berry ripening onset: new insights into ABA signaling network. Front Plant Sci.

[33] Wu G, Park MY, Conway SR, Wang JW, Weigel D, Poethig RS (2009). The sequential action of miR156 and miR172 regulates developmental timing in *Arabidopsis*. Cell.

[34] Sun X, Korir NK, Han J, Shangguan L-F, Kayesh E, Leng X-P, Fang J-GJM (2012). Characterization of grapevine microR164 and its target genes. Mol Biol Rep.

[35] Ferreira e Silva GF, Silva EM, Azevedo Mda S, Guivin MA, Ramiro DA, Figueiredo CR, Carrer H, Peres LE, Nogueira FT (2014). microRNA156-targeted SPL/SBP box transcription factors regulate tomato ovary and fruit development. Plant J.

[36] Bi F, Meng X, Ma C, Yi G (2015). Identification of miRNAs involved in fruit ripening in Cavendish bananas by deep sequencing. BMC Genomics.

[37] Wang Q-H, Zhao C, Zhang M, Li Y-Z, Shen Y-Y, Guo J-X (2017). Transcriptome analysis around the onset of strawberry fruit ripening uncovers an important role of oxidative phosphorylation in ripening. Sci Rep.

[38] Pelsy F (2010). Molecular and cellular mechanisms of diversity within grapevine varieties. Heredity.

[39] Sunkar R, Kapoor A, Zhu J-K (2006). Posttranscriptional induction of two Cu/Zn superoxide dismutase genes in *Arabidopsis* is mediated by downregulation of miR398 and important for oxidative stress tolerance. Plant Cell.

[40] Tang J, Chu C (2017). MicroRNAs in crop improvement: fine-tuners for complex traits. Nat Plants.

[41] Candar-Cakir B, Arican E, Zhang B (2016). Small RNA and degradome deep sequencing reveals drought-and tissue-specific micrornas and their important roles in drought-sensitive and drought-tolerant tomato genotypes. Plant Biotechnol J.

[42] Moxon S, Jing R, Szittya G, Schwach F, Rusholme Pilcher RL, Moulton V, Dalmay T (2008). Deep sequencing of tomato short RNAs identifies microRNAs targeting genes involved in fruit ripening. Genome Res.

[43] López-Vidal O, Camejo D, Rivera-Cabrera F, Konigsberg M, Villa-Hernández J, Mendoza-Espinoza J, Pérez-Flores L, Sevilla F, Jiménez A, de León-Sánchez FD (2016). Mitochondrial ascorbate–glutathione cycle and proteomic analysis of carbonylated proteins during tomato (*Solanum lycopersicum*) fruit ripening. Food Chem.

[44] Jimenez A, Hernandez JA, del Río LA, Sevilla F (1997). Evidence for the presence of the ascorbate-glutathione cycle in mitochondria and peroxisomes of pea leaves. Plant Physiol.

[45] Zhang X, Zou Z, Gong P, Zhang J, Ziaf K, Li H, Xiao F, Ye Z (2011). Over-expression of microRNA169 confers enhanced drought tolerance to tomato. Biotech Letters.

[46] Hong YG, Jackson S (2015). Floral induction and flower formation-the role and potential applications of miRNAs. Plant Biotechnol J.

[47] Reyes JL, Chua NH (2007). ABA induction of miR159 controls transcript levels of two MYB factors during *Arabidopsis* seed germination. Plant J.

[48] Sun Liang, Zhang Mei, Ren Jie, Qi Jianxun, Zhang Guojun, Leng Ping (2010). Reciprocity between abscisic acid and ethylene at the onset of berry ripening and after harvest. BMC Plant Biology.

[49] Csukasi F, Donaire L, Casañal A, Martínez-Priego L, Botella MA, Medina-Escobar N, Llave C, Valpuesta V (2012). Two strawberry miR159 family members display developmental-specific expression patterns in the fruit receptacle and cooperatively regulate Fa-GAMYB. New Phytol.

[50] Linder P, Jankowsky E (2011). From unwinding to clamping - the DEAD box RNA helicase family. Nat Rev Mol Cell Biol.

[51] Chen J, Wan S, Liu H, Fan S, Zhang Y, Wang W, Xia M, Yuan R, Deng F, Shen F (2015). Overexpression of an *Apocynum venetum* DEAD-box helicase gene (*AvDH*1) in cotton confers salinity tolerance and increases yield in a saline field. Front Plant Sci.

[52] Luo Y, Liu YB, Dong YX, Gao XQ, Zhang XS (2009). Expression of a putative alfalfa helicase increases tolerance to abiotic stress in *Arabidopsis* by enhancing the capacities for ROS scavenging and osmotic adjustment. J Plant Physiol.

[53] Mittler R, Vanderauwera S, Gollery M, Van Breusegem F (2004). Reactive oxygen gene network of plants. Trends Plant Sci.

[54] Kant P, Kant S, Gordon M, Shaked R, Barak S (2007). STRESS RESPONSE SUPPRESSOR1 and STRESS RESPONSE SUPPRESSOR2, two DEAD-box RNA helicases that attenuate *Arabidopsis* responses to multiple abiotic stresses. Plant Physiol.

[55] Raja V, Majeed U, Kang H, Andrabi KI, John R (2017). Abiotic stress: interplay between ROS, hormones and MAPKs. Environ Exp Bot.

[56] Pilati S, Perazzolli M, Malossini A, Cestaro A, Demattè L, Fontana P, Dal Ri A, Viola R, Velasco R, Moser C (2007). Genome-wide transcriptional analysis of grapevine berry ripening reveals a set of genes similarly modulated during three seasons and the occurrence of an oxidative burst at veraison. BMC Genomics.

[57] Pilati S, Brazzale D, Guella G, Milli A, Ruberti C, Biasioli F, Zottini M, Moser C (2014). The onset of grapevine berry ripening is characterized by ROS accumulation and lipoxygenase-mediated membrane peroxidation in the skin. BMC Plant Biol.

[58] Baek D, Kim MC, Chun HJ, Kang S, Park HC, Shin G, Park J, Shen M, Hong H, Kim W-Y (2013). Regulation of miR399f transcription by AtMYB2 affects phosphate starvation responses in *Arabidopsis*. Plant Physiol.

[59] Bari R, Pant BD, Stitt M, Scheible W-R (2006). PHO2, microRNA399, and PHR1 define a phosphate-signaling pathway in plants. Plant Physiol.

[60] Okamoto T, Taguchi M, Osaki T, Fukumoto S, Fujita T (2014). Phosphate enhances reactive oxygen species production and suppresses osteoblastic differentiation. J Bone Miner Metab.

[61] Jia F, Wan X, Zhu W, Sun D, Zheng C, Liu P, Huang J (2015). Overexpression of mitochondrial phosphate transporter 3 severely hampers plant development through regulating mitochondrial function in Arabidopsis. PLoS One.

[62] Grimplet J, Agudelo-Romero P, Teixeira RT, Martinez-Zapater JM, Fortes AM (2016). Structural and functional analysis of the GRAS gene family in grapevine indicates a role of GRAS proteins in the control of development and stress responses. Front Plant Sci.

[63] Fortes AM, Agudelo-Romero P, Silva MS, Ali K, Sousa L, Maltese F, Choi YH, Grimplet J, Martinez-Zapater JM, Verpoorte R (2011). Transcript and metabolite analysis in Trincadeira cultivar reveals novel information regarding the dynamics of grape ripening. BMC Plant Biol.

[64] Samad AFA, Sajad M, Nazaruddin N, Fauzi IA, Murad AMA, Zainal Z, Ismail I (2017). MicroRNA and transcription factor: key players in plant regulatory network. Front Plant Sci.

[65] Kumar R (2014). Role of microRNAs in biotic and abiotic stress responses in crop plants. Appl Biochem Biotechnol.

[66] Lei K-J, Lin Y-M, Ren J, Bai L, Miao Y-C, An G-Y, Song C-P (2015). Modulation of the phosphate-deficient responses by microRNA156 and its targeted SQUAMOSA PROMOTER BINDING PROTEIN-LIKE 3 in Arabidopsis. Plant Cell Physiol.

[67] Yamasaki H, Hayashi M, Fukazawa M, Kobayashi Y, Shikanai T (2009). SQUAMOSA promoter binding protein–like7 is a central regulator for copper homeostasis in *Arabidopsis*. Plant Cell.

[68] Hsieh L-C, Lin S-I, Shih AC-C, Chen J-W, Lin W-Y, Tseng C-Y, Li W-H, Chiou T-J (2009). Uncovering small RNA-mediated responses to phosphate deficiency in Arabidopsis by deep sequencing. Plant Physiol.

[69] Li W-X, Oono Y, Zhu J, He X-J, Wu J-M, Iida K, Lu X-Y, Cui X, Jin H, Zhu J-K (2008). The Arabidopsis NFYA5 transcription factor is regulated transcriptionally and posttranscriptionally to promote drought resistance. Plant Cell.

[70] Yuan Y, Fang L, Karungo SK, Zhang L, Gao Y, Li S, Xin H (2016). Overexpression of VaPAT1, a GRAS transcription factor from Vitis amurensis, confers abiotic stress tolerance in Arabidopsis. Plant Cell Rep.

[71] Czikkel BE, Maxwell DP (2007). NtGRAS1, a novel stress-induced member of the GRAS family in tobacco, localizes to the nucleus. J Plant Physiol.

[72] Coombe B (1995). Growth stages of the grapevine: adoption of a system for identifying grapevine growth stages. Aust J Grape Wine Resh.

[73] Rienth M, Torregrosa L, Ardisson M, De Marchi R, Romieu C (2014). Versatile and efficient RNA extraction protocol for grapevine berry tissue, suited for next generation RNA sequencing. Aust J Grape Wine Res.

[74] Wen M, Shen Y, Shi S, Tang T (2012). miREvo: an integrative microRNA evolutionary analysis platform for next-generation sequencing experiments. BMC Bioinformatics.

[75] Friedlander MR, Mackowiak SD, Li N, Chen W, Rajewsky N (2012). miRDeep2 accurately identifies known and hundreds of novel microRNA genes in seven animal clades. Nucleic Acids Res.

[76] Wu HJ, Ma YK, Chen T, Wang M, Wang XJ (2012). PsRobot: a web-based plant small RNA meta-analysis toolbox. Nucleic Acids Res.

[77] Yu G, Wang L, Han Y, He Q (2012). clusterProfiler: an R package for comparing biological themes among gene clusters. MICS.

