# Supplementary figures and images for: MicroRNA profiling analysis of developing berries for ‘Kyoho’ and its early-ripening mutant during berry ripening
Source: BMC Plant Biol. 2018 Nov 16;18:285. doi: 10.1186/s12870-018-1516-x (PMC6240241; doi:10.1186/s12870-018-1516-x)

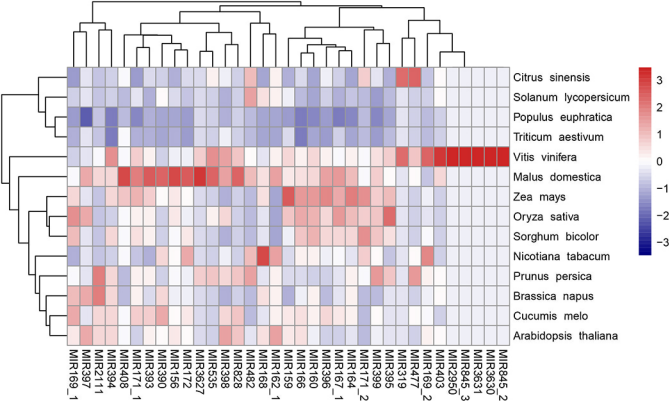

Supplement: Supplementary file 5 — Figure S1. Deeply sequence conserved and previously reported miRNA families detected from developing berries of ‘Fengzao’ and ‘Kyoho’. miRNA families (columns) are conserved between plants families (rows) for plant species represented in miRBase release 21. The bar represents the scale of the numbers of the miRNAs after the Z-score standardization. (PDF 690 kb) [file 12870_2018_1516_MOESM5_ESM.pdf]

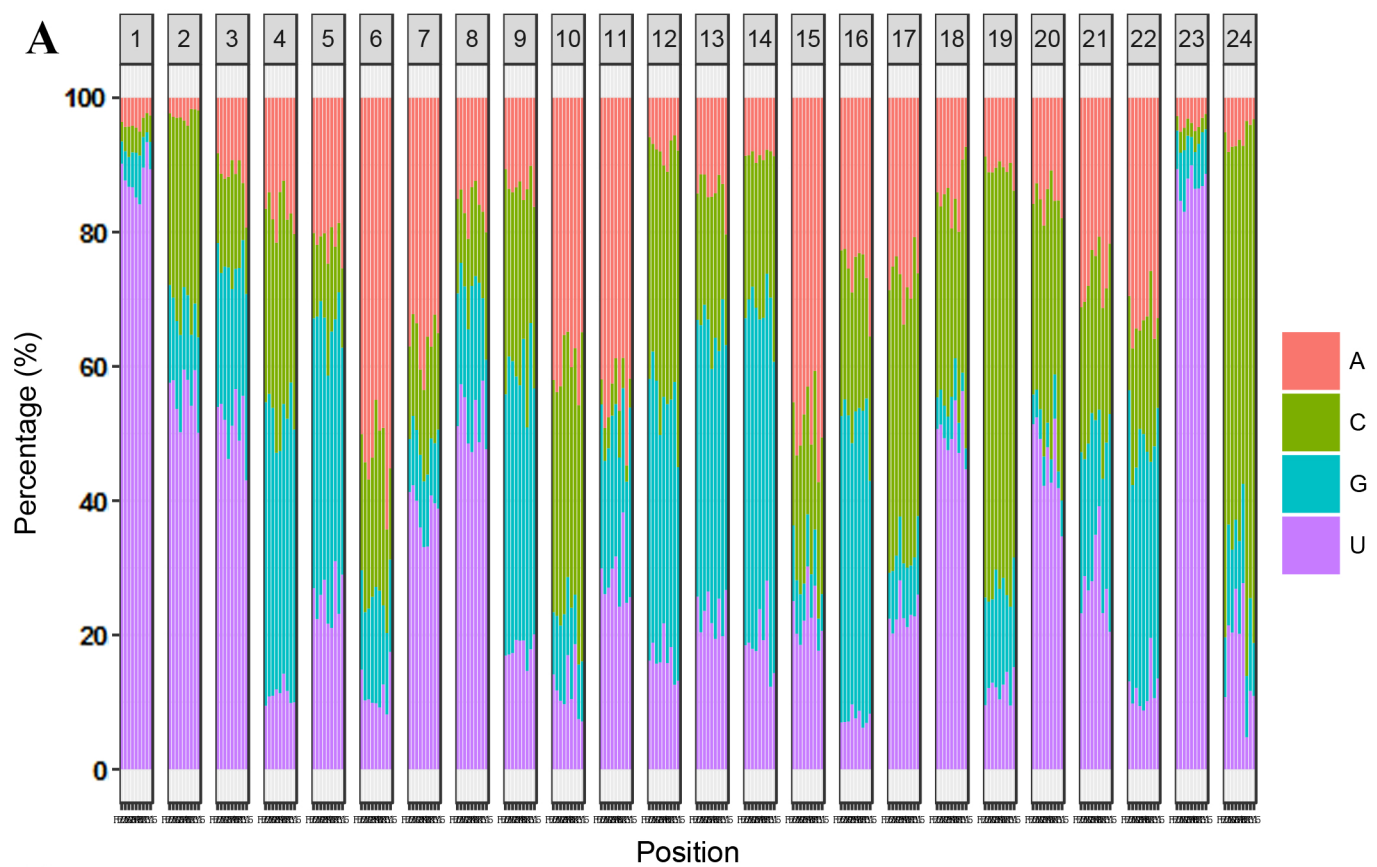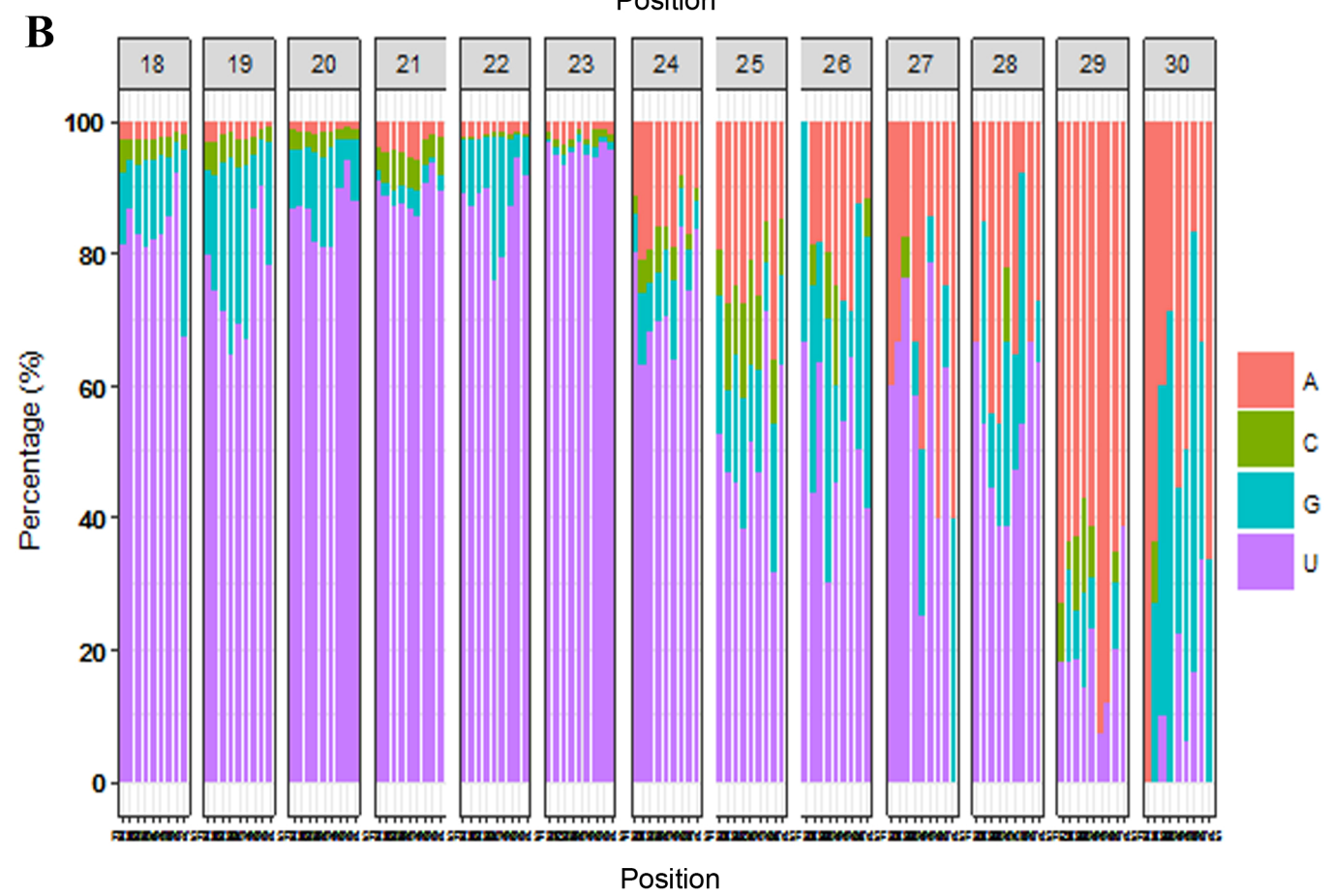

Supplement: Supplementary file 7 — Figure S2. miRNA variants and their nucleotide bias position. A: MiRNA nucleotide bias at each miRNA position. B: First nucleotide bias for the first position of 18- to 30-nt miRNAs. Relative nucleotide bias at each miRNA position compared with the total RNA. (PDF 7480 kb) [file 12870_2018_1516_MOESM7_ESM.pdf]

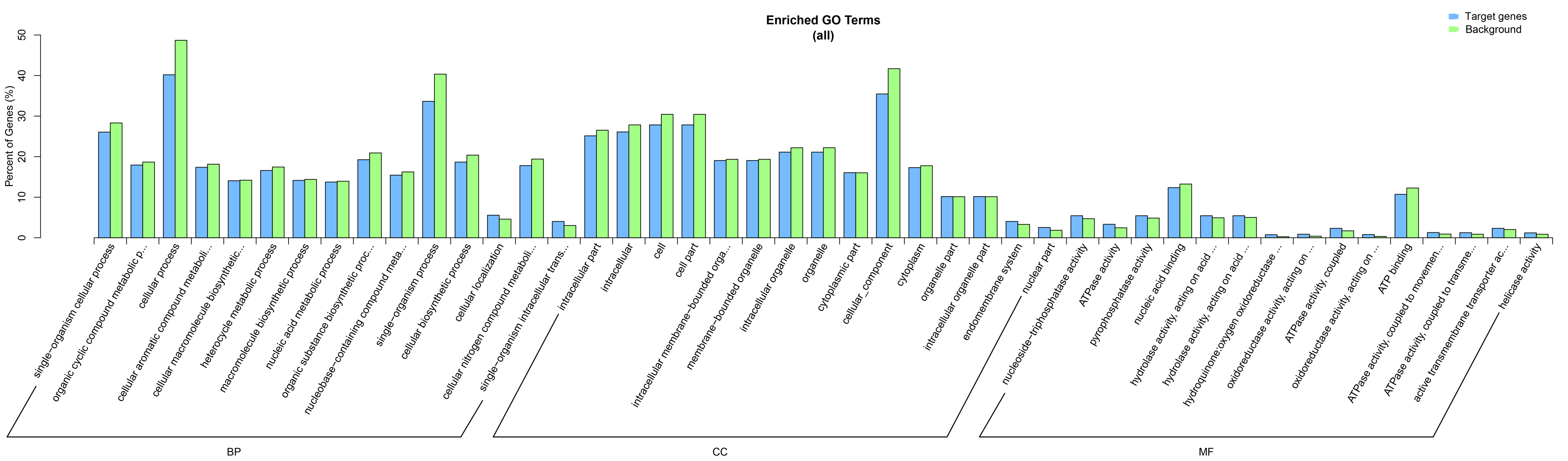

Supplement: Supplementary file 8 — Figure S3. GO categories and distribution of miRNA targets gene of all the miRNAs identified in this study. The left-hand-side scale is the percent of the targeted gene numbers corresponding to the GO terms. (PDF 2795 kb) [file 12870_2018_1516_MOESM8_ESM.pdf]

# Statistics of Pathway Enrichment

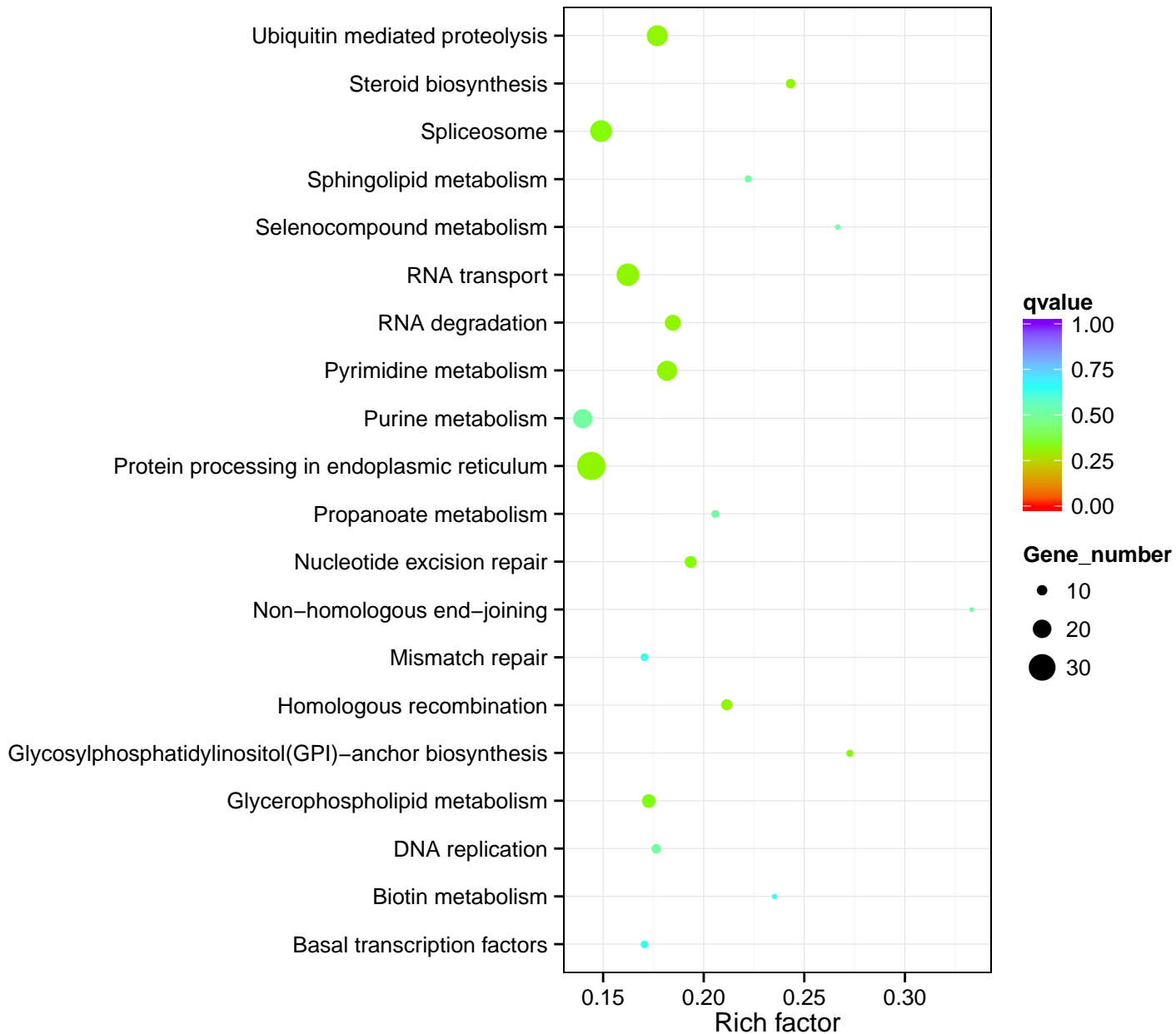

Supplement: Supplementary file 9 — Figure S4. KEGG analysis of the 20 most enriched pathways. The coloring of the q-values indicates the significance of the rich factor. The circle indicates the target genes that are involved, and the size is proportional to the gene numbers. The x-axis represents name of enrichment pathway. The Y-axis represents rich factor. (PDF 6 kb) [file 12870_2018_1516_MOESM9_ESM.pdf]
